# The Impact of Parental Incarceration on Sport Participation Trajectories from Adolescence to Young Adulthood

**DOI:** 10.3390/ijerph17145229

**Published:** 2020-07-20

**Authors:** Ji-Won Park, Jongnam Hwang, Chung Gun Lee, Hyoyeon Ahn, Hanbeom Kim

**Affiliations:** 1 Department of Taekwondo, College of Physical Education and Science, Woo-Suk University Wanju-gun, Jeollabuk-do 55338, Korea; luvprof@naver.com; 2 Division of Social Welfare & Health Administration, College of Social Sciences, Wonkwang University, Iksan 54538, Korea; jiho34@wku.ac.kr; 3 Department of Physical Education, College of Education, Seoul National University, Seoul 08826, Korea; cgl81@snu.ac.kr (C.G.L.); wittymr.ahn@gmail.com (H.A.)

**Keywords:** sport participation, parental incarceration, group-based trajectory modeling, adolescents, young adults

## Abstract

Given the potential negative effects of parental incarceration on millions of people, it is critical to examine the possible short- and long-term effects of parental incarceration on individuals. This study examines the effect of parental incarceration on the sports participation trajectories of children ranging from adolescence to young adulthood. Group-based trajectory models were set up using SAS analytics software to examine how parental incarceration affects the sports participation trajectories of children from adolescence to young adulthood. Data were drawn from the first four waves of the National Longitudinal Study of Adolescent Health in the United States (*N* = 6504). Neither paternal nor maternal incarceration had any significant effect on the trajectories of male participants. On the other hand, females who experienced father incarceration were more likely to be in the low-stable versus high-decreasing group (coefficient = −0.721, *p* < 0.05). Based on the results of this study, we conclude that programs promoting sports participation are needed for females who have experienced paternal incarceration. The results of this study also suggest that group-based trajectory modeling is a useful technique to examine the trajectories of sports participation from adolescence through to young adulthood.

## 1. Introduction

Participation in sports is “an investment that works” to promote regular physical activity [1]. Indeed, the physical health benefits of regular physical activity are almost identical to those of participating in sporting activities. Sport participation has been shown to improve musculoskeletal fitness, metabolic stability, and cardiovascular health [2,3,4,5]. Participation in sports has also been shown to promote psychosocial health by promoting social bonding, promoting cognitive function, and reducing emotional distress [6,7,8]. People who participated in sports from adolescence through young adulthood showed higher self-esteem and happiness and more improved psychological well-being than non-participants in [9]. Despite these physical and psychosocial health benefits of sport participation, about 30% of participants drop out from organized youth sport programs every year, and approximately 70% of participants who dropped out were adolescents [6]. High dropout rates for organized youth sport programs underline the need for long-term follow-up studies to understand why and how trajectories of sport participation change from adolescence through young adulthood.

Life-changing events can be defined as “those occurrences, including social, psychological and environmental, which require an adjustment or effect a change in an individual’s pattern of living.” [10]. These life-changing events may also affect an individual’s physical-activity-related behaviors, such as sport participation, outdoor recreation, and exercise, by disturbing an individual’s daily routine or creating emotional distress [11,12]. Since several life-changing events were shown to have short-term effects on physical-activity-related behaviors, future studies are needed to examine the longer-term effects of life-changing events on physical-activity-related behaviors [12].

People who experienced parental imprisonment have been referred to as the “unseen victims of the prison boom” [13] and the “orphans of justice” [14]. These people may experience multiple social and emotional difficulties during their parents’ imprisonment, which may develop into an array of negative outcomes in the long term. There is a link between parental confinement and children’s mental health, drug use, and educational performance. Children with incarcerated parents are at greater risk of engaging in antisocial behavior compared to their peers [15]. The United States has the highest rate of incarceration in the world (756 per 100,000) [16], and more than half of prisoners in the Unites States are parents [1]. Therefore, given the potential negative effects of parental incarceration on millions of people, it is critical to examine possible short- and long-term effects of parental incarceration on individuals. The experience of parental incarceration has been shown to be associated with residential mobility, economic disadvantage, stigma, maltreatment, primary caregiver disruptions, and exposure to non-biological parent figures [17,18,19,20,21,22]. These associations may be caused by the absence of stable, safe, and nurturing environments during parental incarceration. Several studies also found undesirable long-term effects of parental incarceration on the behavior of offspring. In a longitudinal study, boys who experienced parental incarceration before age 10 years were nearly two times more likely to engage in antisocial behavior up to age 48 years compared to boys without experience of parental incarceration [23,24,25]. Another longitudinal study also found that maternal incarceration is positively related to offspring criminal behavior during adulthood [26]. Likewise, it is possible that parental incarceration may also limit offspring from engaging in physical-activity-related behaviors because parental support is one of the crucial factors that determine these behaviors in offspring [27]. Despite this possibility, none of the studies investigated physical-activity-related behaviors among individuals who have experienced parental incarceration during key developmental periods. There is only one study showing that middle-age women who had an incarcerated family member were less likely to be physically active than women who did not have an incarcerated relative [28].

Although some evidence suggests that a child’s participation in sports can be affected by the incarceration of a parent, the extent and nature of this relationship across the lifespan is unclear. The National Longitudinal Study of Adolescent Health (Add Health), which prospectively followed a nationally representative sample of high school and middle school students in the United States, provided researchers an opportunity to examine the effects of life-change events on participation in sports from adolescence to young adulthood. Based on the research gaps identified from current literature, this study examines the effect of parental incarceration on the sports participation trajectories of children ranging from adolescence to young adulthood..

## 2. Materials and Methods

### 2.1. Data

The data for this study were collected from Add Health, a four-wave longitudinal study that tracked nationally representative samples of middle and high school students from the 7th to 12th grade in the United States. The initial sampling involved all public and private high schools, with more than 30 students being included from each school. These schools were classified according to ethnicity, size, region, urbanicity, and school type. Systematic random sampling was used to choose 80 high schools, 70% of which were recruited to the study. Middle schools that sent graduates to selected high schools were also recruited to the study. The final sample included 134 middle schools and high schools. Students were randomly selected from each school after stratification by age and grade based on official school rosters. From April to December 1995, in-home surveys were conducted for wave 1 (*N* = 20,745). Approximately 1 year later, a follow-up survey (wave 2) was conducted (*N* = 14,738). Six and twelve years after wave 1, waves 3 (*N* = 15,197) and 4 (*N* = 15,701), respectively, were conducted. Additional data and information regarding Add Health can be found online [29]. This study used public data sets for waves 1 to 4 (*N* = 6504). The Add Health data used in this study conforms to the University of North Carolina School of Public Health’s ethical guidelines and does not include personal information.

### 2.2. Measures

#### 2.2.1. Dependent Variable

Participation in sports during wave 1 and 2 was assessed by the following question: “How many times did you play an active sport (baseball, softball, basketball, soccer, swimming, or football), within the past week?” Since sport participation during waves 3 and 4 were assessed with two questions, “How many times have you participated in individual sports (running, wrestling, swimming, cross-country skiing, cycle racing, or martial arts) within the past week?” and “How many times have you participated in active team sports (football, soccer, basketball, lacrosse, rugby, field hockey, or ice hockey) within the past week?”, responses regarding both team and individual sports were combined, giving the total number of times students participated in sports in the past week. Participants who participated in sports more than five times during the week were defined as active participators.

#### 2.2.2. Independent Variable

Parental incarceration was measured during wave four by means of in-home surveys by asking the following two questions; “At what age were you when your biological mother went to prison for the first time?” and “At what age were you when your biological father went to prison for the first time?”. When the participants’ mother or father were in prison before wave 1 of the survey, participants were defined as those who had an incarcerated mother or father.

### 2.3. Statistical Analysis

For this study, the influence of parental incarceration on the trajectories of sports participation, from adolescence to young adulthood, was investigated using group-based trajectory modeling. The group-based trajectory modeling summarizes large amounts of data in a comprehensible fashion by segmenting the data into several trajectory groups based on a participant s probability of being in a certain group. Participation in sports was considered a binary variable, for which binary logit models estimated trajectories via the SAS TRAJ procedure [30]. The wave was used as a time scale, centered at wave 1. Based on non-statistical [31] and statistical [32] criteria, the measurement and shape of each trajectory group were chosen. The Bayesian information criterion (BIC), percentages of observations assigned to each group, and average posterior probabilities of group assignment were used to identify the most useful and parsimonious model [31]; this decision was also informed by subjective judgment. After the best model was selected, independent variables for trajectory group assignment were added. The goal was to examine the effect of paternal and maternal incarceration before wave 1 on trajectories of sports participation [33]. The participant characteristics data are provided as frequency, percent (for categorical variables), mean, and standard deviation values. The chi-squared test and analysis of variance (ANOVA) were used to compare categorical and continuous variables, respectively, between the trajectory groups. All data were analyzed using SAS software (version 9.4; SAS Institute Inc., Cary, NC, USA).

## 3. Results

### 3.1. Descriptive Statistics

Table 1 shows the characteristics of the male participants by sports participation trajectory group. The mean age of the male participants in wave 1 was 15.61 years. Male participants were divided into three sports participation trajectory groups (i.e., high-stable, high-decreasing, and low-stable groups). For each wave, the prevalence of paternal and maternal incarceration was similar among the groups. Table 2 shows the characteristics of female participants by sports participation trajectory group. Unlike male groups, there were two sports participation trajectory groups for female participants (i.e., high-decreasing and low-stable groups). The mean age of female participants in wave 1 (15.46 years) was the same as that of male participants. The prevalence of paternal incarceration was higher in the low-stable versus high-decreasing group, while the prevalence of maternal incarceration was similar between the two trajectory groups.

### 3.2. Group-Based Trajectory Modeling

**Figure 1:**
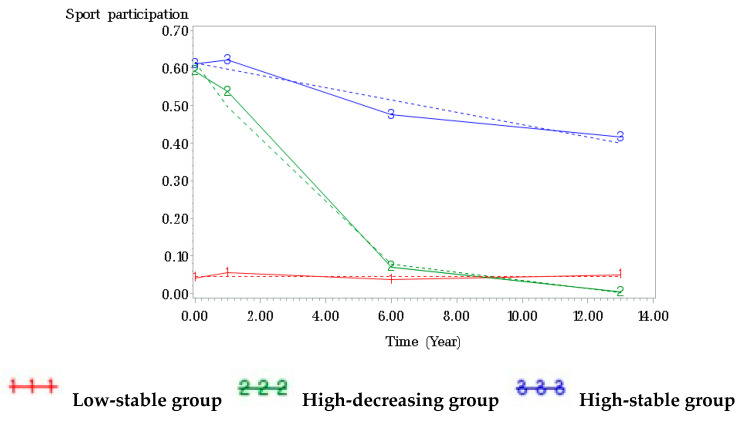
Trajectories of sport participation from adolescence through young adulthood among male participants. Note. Solid line represents average data; dashed line represents mean trajectories.

**Figure 2:**
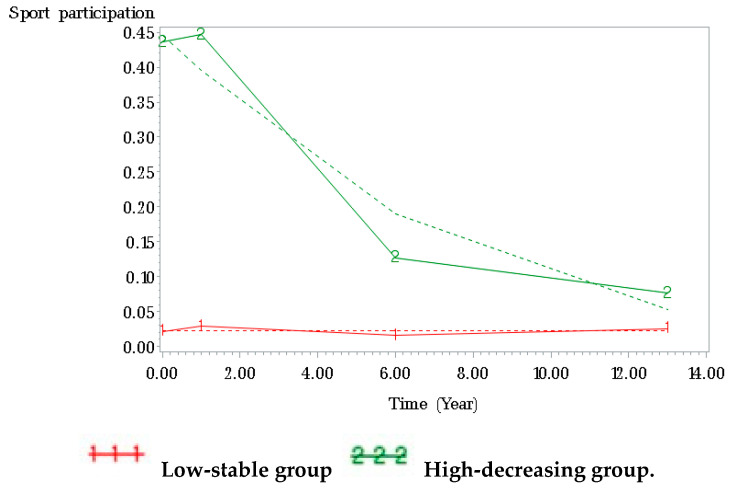
Trajectories of sport participation from adolescence through young adulthood among female participants. Note. Solid line represents average data; dashed line represents mean trajectories.

A three-group trajectory model provided the best fit for the sports participation data of the males (Figure 1). About half (54.12%) of all male participants were assigned to the low-stable group. The remaining male participants (45.88%) started with a high probability of participating in sports, with 10.20% maintaining that high probability into young adulthood (high-stable group) and 35.68% being less likely to do sports over time (high-decreasing group). A two-group trajectory model was the best fit for the sports participation data of the females (Figure 2). Of female participants, 24.70% had a high probability of participation in sports in adolescence, which decreased until they reached young adulthood. The remaining female participants (75.30%) were assigned to the low-stable group.

Table 3 and Table 4 show the influence of parental incarceration on the sports participation trajectories of males and females, respectively. Neither paternal nor maternal incarceration had any significant effect on the trajectories of the male participants. On the other hand, paternal incarceration affected the trajectories of the female participants. Females who experienced paternal incarceration were more likely to be in the low-stable versus high-decreasing group (coefficient = −0.721, *p* < 0.05).

## 4. Discussion

A growing body of research has highlighted various factors influencing participation in sports among young people, where parental support is one of the most crucial determinants [34]. Habitual physical activity that is established at a younger age can persist into adulthood, and it is important for adolescents to receive emotional and social support from their parents [27,35,36,37]. In this study, we assessed whether paternal or maternal incarceration affected sports participation among their offspring. To our knowledge, this is the first study to examine the effect of parental imprisonment on the sports participation trajectories of young people from adolescence through to young adulthood, using group-based trajectory modeling.

Our group-based trajectory model classified males into three trajectory groups (i.e., low-stable, high-decreasing, and high-stable). The largest group (54.12%) had a low probability of sports participation throughout the period from adolescence to young adulthood. The other groups started with a high probability of participation in sports activities during adolescence, but differences in the sports participation rate became apparent over time; one group showed a consistently high probability of sports participation across the study period, while the other group showed less participation in sports activities. Meanwhile, females were divided into only two trajectory groups. Most of the females (75.30%) had a low probability of participation in sports-related activities from adolescence to young adulthood; the other group (24.70%) had a high probability, but this decreased over time. One interesting finding is that there was no “low-increasing group” for either the males or females. This is supported by existing studies suggesting that the overall prevalence of physical activities and sports participation decreases during the transition from adolescence to adulthood; therefore, it is important to provide more opportunities for such activities, as well as activity-promoting environments for young people [38,39,40]. For instance, physical education programs aimed at young people should be implemented at the school and community levels, such that participation in sports increases in both male and female youths. Factors associated with engagement in physical and sports-related activities by youths must be better understood, particularly for females, given the lack of a high-stable group in this study.

Our results also showed that females whose fathers were incarcerated during their youth were less likely to participate in sport activities, suggesting that the role of fathers may impact offspring sports participation. In contrast, no association between participation in sports activities and parental incarceration was observed in males.

Our findings are consistent with previous studies highlighting that the role of parents is one of the most important determinants of a child’s level of activity. Previous studies have suggested that, in terms of the physical activity of their children, parents serve not only as role models for their offspring, but also as “gatekeepers”, by encouraging their children to participate in sports events or registering them in various exercise or sports classes [41,42,43]. In addition, parental support enables their children to have access to exercise resources or events, implying that the absence of parents in early life may preclude offspring participating in physical activity or sports [44]. Thus, children with parents who guide them toward sports-related activities at an early age will be more likely to maintain positive habits regarding physical activities, and may continue participating in sports or physical activities into adulthood. In fact, parents can influence their child’s participation in sports-related activities in various phases of their life [45]. For example, previous studies have consistently reported that parental support, in addition to opportunities to exercise, were correlated with an adolescent’s physical activity level [44,46]. Thus, parental absence (i.e., low levels of parental monitoring and supervision) may increase the likelihood of behaviors associated with non-participation in sports activities [47].

## 5. Conclusions

It is worth noting that parental incarceration affected only female participation in sports activities. A plausible explanation for this concerns the role of the father in the development of children, especially among females. The female youths in this study were less likely to participate in sports activities in general, which implies that they might not actively engage in physical activities outside of the school environment. The influence of fathers on the physical activity of their children is likely greater in the context of after school and weekend activities [48]. Taken together, our results suggest that absence of the father during adolescence may result in low motivation to participate in physical or sports activities among females; this finding is in line with the existing literature, highlighting the pivotal role of parents in physical or sports activities among their offspring.

This study had four important limitations. First, since highly detailed information about sports participation was not available in the Add Health data set, individual and team sports could not be distinguished. To better understand the influence of parental incarceration on sports participation, future studies should include more precise measures of sports participation. Second, the small number of data waves may have affected the group membership data. Only linear trajectory shapes were statistically significant, possibly because the Add Health data had only four waves. Third, all of the data in this study were from self-report questionnaires, which can introduce response and recall biases. Fourth, since public data were used in this research, it was difficult to control various factors affecting adolescents’ sport participation.

Despite these limitations, the findings of this study are important for understanding how parental incarceration affects sports participation trajectories among young people, from adolescence through to young adulthood. Programs to promote sports participation for females who have experienced paternal incarceration are needed. The results of this study also suggest that group-based trajectory modeling is a useful technique to examine the trajectories of sports participation from adolescence through to young adulthood.

## Figures and Tables

**Table 1:** Characteristics of male participants by sport participation trajectory group (*N* = 3147).

Characteristics	Total Sample	Sport Participation Trajectory Groups			*p*-Value
		Low-Stable	High-Decreasing	High-Stable	
Mean age at wave 1 (SD)	15.61 (1.79)	15.92 (1.79)	15.27 (1.71)	15.18 (1.77)	<0.0001
Paternal incarceration before wave 1 (%)	166 (7.43)	85 (7.00)	66 (8.82)	15 (5.54)	0.1452
Maternal incarceration before wave 1 (%)	34 (1.45)	16 (1.27)	12 (1.51)	6 (2.11)	0.5532
Sport participation≥ 5 times per week (%)					
Wave 1	1067 (33.94)	0 (0.00)	849 (75.60)	218 (68.13)	<0.0001
Wave 2	781 (33.75)	0 (0.00)	607 (65.48)	174 (67.18)	<0.0001
Wave 3	273 (12.17)	46 (3.87)	0 (0.00)	227 (77.74)	<0.0001
Wave 4	218 (9.27)	61 (4.81)	0 (0.00)	157 (54.70)	<0.0001
Total (%)	3147	1703 (54.12)	1123 (35.68)	321 (10.20)	

Note. Missing data were excluded in calculating the percentage.

**Table 2:** Characteristics of female participants by sport participation trajectory group (*N* = 3356).

Characteristics	Total Sample	Sport Participation Trajectory Groups		*p*-Value
		Low-Stable	High-Decreasing	
Mean age at wave 1 (SD)	15.46 (1.78)	15.72 (1.76)	14.69 (1.62)	<0.0001
Paternal incarceration before wave 1 (%)	193 (7.35)	158 (8.04)	35 (5.28)	0.0183
Maternal incarceration before wave 1 (%)	54 (1.96)	41 (1.99)	13 (1.88)	0.8510
Sport participation ≥ 5 per week (%)				
Wave 1	515 (15.35)	0 (0.00)	515 (62.12)	<0.0001
Wave 2	422 (16.75)	0 (0.00)	422 (58.29)	<0.0001
Wave 3	134 (5.12)	0 (0.00)	134 (19.45)	<0.0001
Wave 4	115 (4.17)	62 (3.00)	53 (7.65)	<0.0001
Total (%)	3356	2527 (75.30)	829 (24.70)	

Note. Missing data were excluded in calculating the percentage.

**Table 3:** The effects of parental incarceration on sport participation trajectories among male participants (*N* = 3147).

Group	Parameter	Estimate (*SE*)	
Low-stable	Reference group		
High-decreasing	Constant	−0.337	(0.150) *
	Maternal incarceration before wave 1	0.390	(0.694)
	Paternal incarceration before wave 1	0.405	(0.263)
High-stable	Constant	−1.027	(0.224) **
	Maternal incarceration before wave 1	0.800	(0.728)
	Paternal incarceration before wave 1	−0.221	(0.380)

* *p* < 0.05, ** *p* < 0.001.

**Table 4:** The effects of parental incarceration on sport participation trajectories among female participants (*N* = 3356).

Group	Parameter	Estimate (*SE*)	
Low-stable	Reference group		
High-decreasing	Constant	−0.745	(0.129) **
	Maternal incarceration before wave 1	−0.061	(0.521)
	Paternal incarceration before wave 1	−0.721	(0.295) *

* *p* < 0.05, ** *p* < 0.001.
